# Future Therapeutic Directions for Smac-Mimetics

**DOI:** 10.3390/cells9020406

**Published:** 2020-02-11

**Authors:** Emma Morrish, Gabriela Brumatti, John Silke

**Affiliations:** 1 Inflammation Division, Walter and Eliza Hall Institute of Medical Research, Melbourne VIC 3052, Australia; morrish.e@wehi.edu.au (E.M.); brumatti@wehi.edu.au (G.B.); 2 Department of Medical Biology, University of Melbourne, Melbourne VIC 3010, Australia

**Keywords:** Smac/DIABLO, Smac-Mimetics, IAPs, TNF, cell death, cancer

## Abstract

It is well accepted that the ability of cancer cells to circumvent the cell death program that untransformed cells are subject to helps promote tumor growth. Strategies designed to reinstate the cell death program in cancer cells have therefore been investigated for decades. Overexpression of members of the Inhibitor of APoptosis (IAP) protein family is one possible mechanism hindering the death of cancer cells. To promote cell death, drugs that mimic natural IAP antagonists, such as second mitochondria-derived activator of caspases (Smac/DIABLO) were developed. Smac-Mimetics (SMs) have entered clinical trials for hematological and solid cancers, unfortunately with variable and limited results so far. This review explores the use of SMs for the treatment of cancer, their potential to synergize with up-coming treatments and, finally, discusses the challenges and optimism facing this strategy.

## 1. The Relevance of Programmed Cell Death in Cancer

One of the hallmarks of cancer is failure to undergo genetically programmed cell death in response to signals that would normally promote a suicide response in untransformed cells. Numerous programmed cell death mechanisms have been described and include apoptosis, necroptosis, autophagic cell death, eryptosis, NETosis, ferroptosis, paraptosis and pyroptosis [1]. The most important of these in cancer development are apoptosis and necroptosis. While autophagy very likely helps cancer cells survive low nutrient and other stressful conditions, and there are several well-known instances of autophagic cell death during development, the importance of autophagic cell death in tumor development is still unclear [1,2]. Failure to undergo cell death allows cancer cells to mutate, evolve and proliferate. Therefore, reactivating their ability to commit suicide is an appealing anti-cancer treatment strategy and has been explored in a variety of chemotherapeutic drug approaches. Apoptosis is one type of programmed cell death which occurs under normal physiological conditions and is described as being either intrinsic (mitochondria dependent) or extrinsic (death receptor dependent) [3]. Apoptosis is characterized morphologically by cell shrinkage, membrane blebbing, aggregation of chromatin and formation of apoptotic bodies. The enzymes that cause these phenotypes are cysteine proteases that cleave after an aspartate residue and are therefore known as caspases [4]. In contrast, necrosis, an unregulated form of cell death, leads to loss of cell homeostasis and membrane integrity, resulting in cell rupture [5,6]. Another form of programmed cell death termed necroptosis, which shares features with necrosis such as cell rupture and release of cellular contents, has recently also been explored as an anti-cancer therapeutic strategy [7,8]. Necroptosis is implemented during cellular stress when caspases are inhibited, for example genetically or pharmacologically. The proteins that carry out the necroptotic program are the Receptor-Interacting serine/threonine-Protein Kinases (RIPK) 1 and 3 and the pseudokinase Mixed Lineage Kinase domain-Like protein (MLKL) [9].

## 2. Inhibitor of Apoptosis Proteins

**Figure 1:**
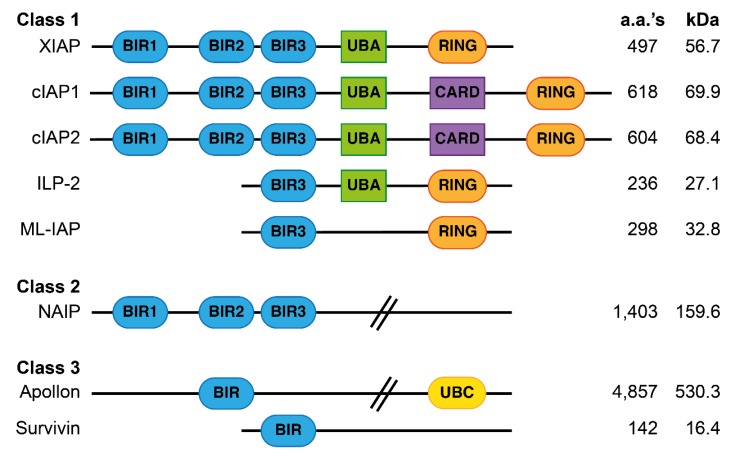
Schematic representation of the structures of the eight-mammalian Inhibitor of APoptosis (IAP) proteins. BIR, Baculovirus IAP repeat domain; UBA, Ubiquitin binding domain; CARD, Caspase recruitment domain; RING, E3-Ligase domain; UBC, E2-Ligase domain; a.a.’s, amino acids; kDa, kilodalton. The position and size of domains are not represented to scale.

The human family of Inhibitor of APoptosis (IAP) proteins regulates cell survival in response to a number of stimuli. The IAP family, defined by the presence of one or more Baculoviral IAP Repeat (BIR) domains, consists of eight members: X-chromosome-linked IAP (XIAP), cellular IAP 1 and 2 (cIAP1 and cIAP2), Melanoma-IAP (ML-IAP), Neuronal-IAP (NAIP), survivin, BIR-containing ubiquitin-conjugating enzyme (Bruce/Apollon) and IAP-Like Protein 2 (ILP-2). Only three of these, cIAP1, cIAP2 and XIAP, have major anti-apoptotic roles and this review therefore focuses on them (Figure 1) [10,11,12]. These three proteins contain three BIR domains, a Really Interesting New Gene (RING)-finger domain, that has Ubiquitin (Ub) ligase (E3) activity, and a UB-Associated (UBA) domain, which enables their interaction with ubiquitylated proteins [13,14]. XIAP is able to bind and inhibit caspases 3, 7 and 9, whilst cIAP1 and 2 inhibit apoptosis induced by members of the Tumor Necrosis Factor (TNF) Super Family (TNFSF), at least in part by regulating RIPK1, a cytoplasmic protein recruited to TNFSF receptors. XIAP is also essential for Nucleotide-binding Oligomerization Domain-containing protein (NOD) signaling and ubiquitylates the NOD binding protein RIPK2, presumably in a similar manner to the way cIAPs ubiquitylate RIPK1 [15,16,17,18,19,20]. However, NOD and RIPK2 do not appear to induce cell death and therefore, XIAP function in this pathway is unlikely to be directly anti-apoptotic.

## 3. TNF Signaling Pathway

**Figure 2:**
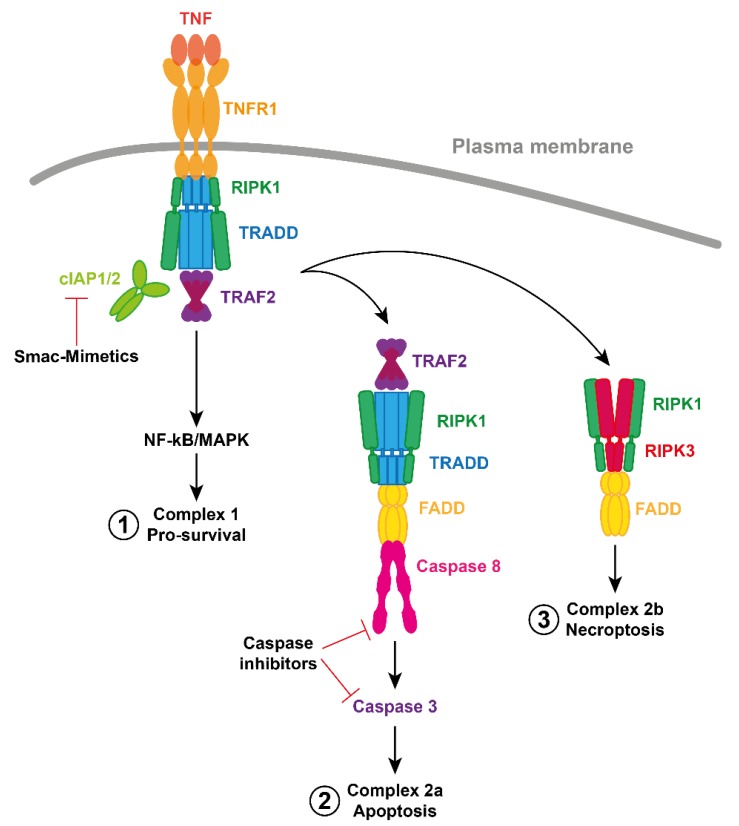
Simplified schematic of TNFR1 Signaling. Formation of complex 1 can lead to activation of canonical NF-κB and MAPK pro-survival signaling. Antagonism (or loss) of cIAP proteins induced by Smac-Mimetics leads to formation of complex 2a, containing TRADD, RIPK1, FADD and caspase-8. Caspase-8 activation and cleavage, and activation of caspase-3, results in apoptosis. Alternatively, if caspases are inhibited, complex 2b can form via a RHIM motif dependent recruitment of RIPK3. RIPK3 auto-phosphorylates and phosphorylates the pseudokinase MLKL. Phosphorylation of MLKL leads to a conformational change, membrane translocation, oligomerization, membrane permeabilization and necroptotic cell death.

TNF is a potent and pleiotropic cytokine responsible for a diverse range of biological functions including inflammation, proliferation and cell death. In most cases, the binding of TNF to its receptor, TNFR1, leads to the recruitment of the TNF Receptor-Associated Death Domain (TRADD) and RIPK1 via their respective death domains. TRADD and RIPK1 can apparently bind TNFR1 at the same time, but the relative contribution of each to the signaling complex may depend upon the cell type [21,22,23]. TRADD can recruit the TNF Receptor-Associated Factor 2 (TRAF2) and cIAP1/2 are bound to TRAF2 via a BIR1 interaction with the coiled coil of a TRAF2 trimer [24,25]. As E3 ubiquitin ligases, cIAP1/2 conjugate components of this complex with ubiquitin. The ubiquitin platform recruits Linear UBiquitin chain Assembly Complex (LUBAC), IκB Kinases (IKKs) and Transforming growth factor beta-Activated Kinase 1 (TAK1), resulting in activation of canonical Nuclear Factor kappa-light-chain-enhancer of activated B cells (NF-κB) and Mitogen-Activated Protein Kinases (MAPKs). This leads to transcriptional upregulation and mRNA stabilization of genes that encode mediators of inflammation and proteins involved in cell survival and proliferation [20,26,27]. Although the details are not fully understood, ubiquitylation and phosphorylation of components within this plasma membrane associated, “complex 1”, limit the formation of the death-inducing complex 2 (Figure 2) [28,29,30,31,32].

Natural antagonists of IAPs, such as second mitochondria-derived activator of caspases (Smac/DIABLO), or HtrA2, bind to the BIR domains of IAPs via their IAP-Binding Motifs (IBMs). Smac and HtrA2 prevent XIAP from binding and inhibiting caspases 3, 7 and 9 [33,34,35,36,37]. IAP antagonists rarely induce XIAP degradation. However, in many cases, they promote cIAP auto-ubiquitylation and proteasomal degradation [17,20]. They do this by unleashing the RING domain of cIAPs from BIR3 inhibition allowing it to dimerize [38,39]. This activation of the E3 ligase function of cIAPs by an IAP antagonist may also result in ubiquitylation of the antagonist but the physiological significance of this is not clear [35,40,41,42]. IAP antagonist induced degradation of cIAPs prevents cIAP-mediated ubiquitylation of components in the TNF signaling pathway and thus converts TNFR1 signaling from pro-survival to pro-apoptotic. In particular, loss of cIAPs allows the formation of a FADD-caspase-8 containing complex 2, leading to caspase-8 activation by oligomerizing in a chain like manner [43,44,45].

In the absence of caspase-8 activity, for example through pharmacological antagonism or genetic deletion, and in the presence of IAP inhibitors (such as Smac-Mimetic drugs), a second cell death mediating complex forms, termed complex 2b. RIPK1 binds with RIPK3 via their Respective Homotypic Interaction Motif (RHIM) domains, leading to auto-phosphorylation and subsequent recruitment of MLKL. RIPK3 phosphorylation and oligomerization of MLKL, leads to its activation and translocation from the cytosol to the plasma membrane, where it disrupts membrane integrity, leading to necroptotic cell death (Figure 2) [26,46,47,48,49].

## 4. Development of Smac-Mimetics

Overexpression of IAPs has been associated with multiple cancers, including hematological and solid cancers, and is indicative of poor prognosis [27,50,51]. Clinically, it was observed that patients expressing higher levels of Smac had a more favorable prognosis, with higher remission rates and longer overall survival [27]. Proof of principle for targeting IAPs was provided by the demonstration that exogenously expressing Smac in resistant neuroblastoma cells sensitized them to TNF-Related Apoptosis-Inducing Ligand (TRAIL) induced apoptosis [52]. Subsequent studies using antisense oligonucleotides against XIAP or synthetic Smac peptides, showed that these also sensitized cancer cells to chemotherapy [53,54]. Together, with other studies, this prompted the pharmaceutical development of small molecule, peptide-like mimetics of Smac, termed Smac-Mimetics (SMs). SMs mimic the minimal N-terminal tetrapeptide (NH_2_-AVPI), that constitutes a significant part of the IBM, which binds to the BIR domains of cIAP1/2 and XIAP [35,55,56]. Many of these mimetic compounds have shown anti-cancer effects in vitro and in vivo, validating the development of clinical SMs [27,57,58,59,60].

**Figure 3:**
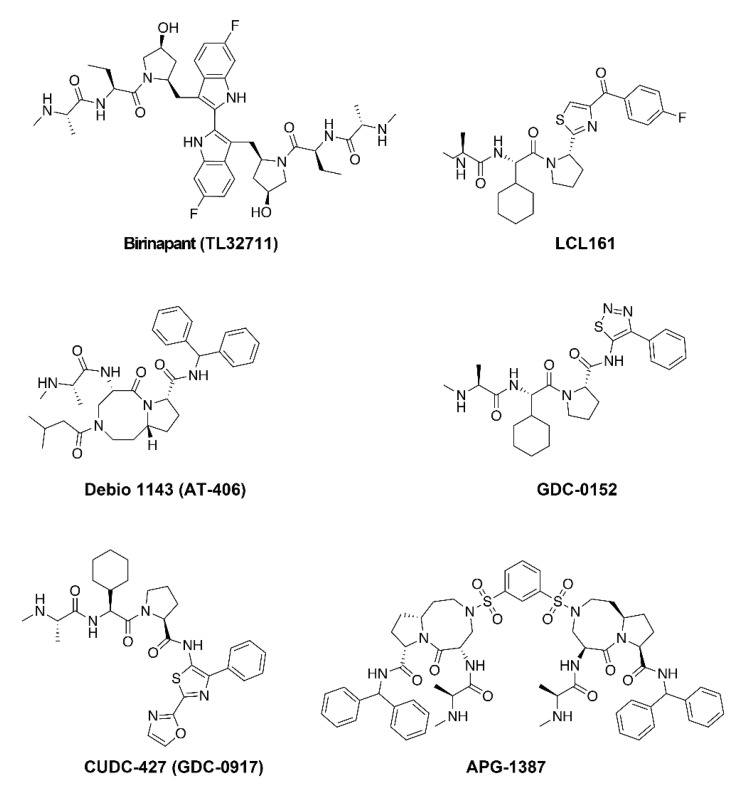
Structures of Smac-Mimetic compounds that have progressed to clinical trials. Note, the structures of HGS1029 (AEG40826) and BI 891065 are not publicly available.

Endogenous Smac homodimerizes through an extensive hydrophobic interface and is bivalent [61]. Potent and selective bivalent SMs, as well as monovalent compounds, have been developed for the clinic. Monovalent SMs have one AVPI-like binding motif whilst bivalent SMs have two. To date, five monovalent compounds, GDC-0152, CUDC-427 (GDC-0917), Debio 1143 (AT-406), LCL161 and BI 891065, and three bivalent compounds, birinapant (TL32711), APG-1387, HGS1029 (AEG40826), have entered clinical trials for the treatment of cancer (Figure 3).

## 5. Smac-Mimetics as Single Agents in Pre-Clinical and Clinical Studies

Triple knock-out *ciap1^−/−^ciap2^−/−^xiap^−/−^* and double knock-out *ciap1^−/−^/ciap2^−/−^* animals are embryonic lethal. In contrast, double knock-out *ciap2^−/−^/xiap^−/−^* are viable [62]. There are two conflicting reports with regard to *ciap1^−/−^/xiap^−/−^* with one strain being embryonic lethal [62] and another viable [63]. At the least, these data suggest that inhibiting all three anti-apoptotic IAPs may be undesirable from a safety perspective. Certainly SMs that inhibit all three with low nanomolar *K*_i_ tend to have a more inflammatory profile and be more toxic to cells than ones with a more restricted profile (Table 1) [50,64,65,66].

### 5.1. Birinapant

Birinapant (previously known as TL32711, Tetralogic Pharmaceuticals) is one of the most clinically progressed SMs [50]. Birinapant’s higher affinity for the BIR3 of cIAP1 (*K*_i_ ~1 nM) than for that of cIAP2 (36 nM) and XIAP (50 ± 23 nM) is likely to contribute to its good safety profile [50,62]. Birinapant’s anti-cancer activity as a single agent has been extensively investigated in vitro and in vivo. Benetatos et al., undertook a large-scale screen in vitro of 111 different malignancies and observed 18 (16%) were sensitive to birinapant single-agent treatment [67]. Interestingly, single-agent birinapant treatment in vivo in 50 patient-derived xenotransplant models of ovarian, colorectal and melanoma cancer, resulted in inhibition of tumor growth in roughly one third [67]. Similarly, although the human melanoma cell lines 451 Lu and 1025 Lu were both resistant to birinapant in vitro, in xenotransplantation models of these cells, treatment with birinapant single agent led to significant slowing of tumor growth for both cell lines [68]. It is possible that this unexpected improvement of efficacy in vivo compared with in vitro, is due to elevated levels of TNF in the microenvironment of melanoma lesions due to chronic inflammation [69]. While TNF has pro-survival effects on melanoma cells, enhancing invasion and migration potential [70], SMs can convert this advantage to a liability. These results highlight that SMs may synergize with an inflammatory environment, whether induced or otherwise, to cause cancer cell death [71,72,73].

A recent analysis of 279 Head and Neck Squamous Cell Carcinoma (HNSCC) tumors by The Cancer Genome Atlas (TCGA) identified roughly thirty percent of HNSCC patients have genomic amplifications of Fas-Associated Death Domain (*FADD*), with a subset of these patients also having amplifications in *BIRC2/cIAP1* or *BIRC3/cIAP2* [74,75]. Birinapant was effective as a single agent both in vitro and in vivo in HNSCC cells overexpressing FADD, with differential expression levels of cIAP1. Interestingly, following overexpression of FADD in the FADD-deficient cell line UM-SCC-38, birinapant treatments were effective at inducing cell death, implicating FADD as an important component in SM mediated killing [74,76]. In Inflammatory Breast Cancer (IBC), overexpression of XIAP has been correlated with acquired therapeutic resistance to apoptotic stimulus such as TRAIL [77]. Single-agent treatment with birinapant in TRAIL resistant IBC cell lines was pro-apoptotic, leading to cell death [78]. The authors proposed that this sensitivity was due to birinapant’s activity towards XIAP, as a related bivalent SM that binds XIAP less potently (*K*_d_ 0.45 and >1 μM respectively) was not as effective at inducing cell death [78]. However, caveats to this conclusion are that cIAP2 binding was not examined and the different physico-chemical properties of the two compounds was not discussed [78].

The first in-human clinical trial with birinapant was in patients with advanced solid tumors or lymphoma (NCT00993239). Birinapant was administered intravenously, with a dose-escalation from 0.18 to 63 mg/m^2^, once a week, every three out of four weeks. The Maximum Tolerated Dose (MTD) was determined as 47 mg/m^2^, with the maximum dose 63 mg/m^2^ having Adverse Effects (AEs) including headache, nausea and vomiting. Intriguingly, 2 out of 3 patients receiving 63 mg/m^2^ presented with Bell’s palsy, a facial nerve paralysis [79]. Although birinapant accumulated in tumor tissue and had on target effects as an IAP inhibitor, no Complete (CR) or Partial Responses (PR) were observed in the 26 patients who were eligible for evaluation. Stable disease was observed in 7 patients (27%) and 2 patients with colorectal cancer demonstrated radiographic evidence of tumor shrinkage [79]. A second Phase I/II clinical trial with birinapant was conducted in patients with relapsed Acute Myeloid Leukemia (AML) or MyeloDysplastic Syndrome (MDS). Birinapant was administered at varying doses (17, 22 or 26 mg/m^2^) and frequency (weekly, twice weekly or three times weekly) [80]. One case of Bell’s palsy was observed at 22 mg/m^2^ dosed twice a week, and the three times a week dosing schedule was abandoned due to feasibility concerns. Best responses included a reduction in bone marrow blasts from 60% to 10% [80]. A Phase II single-agent trial of birinapant in 11 patients with relapsed platinum-resistant or -refractory epithelial ovarian cancer was conducted using the pre-established MTD of 47 mg/m^2^, administered once a week, three out of four weeks (NCT01681368) [79,81]. Similar to previous studies, birinapant demonstrated potent on target inhibition of IAPs, but no clinical benefit was observed, and therefore the study was terminated [81]. Together, these first in-human studies with birinapant indicate that as a single agent, birinapant has some anti-cancer activity but is unlikely to be universally effective in treating cancer. Despite the incidences of Bell’s palsy (which is reversible upon birinapant reduction or withdrawal), the good tolerability profile of birinapant suggests that it has the potential to be combined with chemotherapy and TNF enhancing therapy.

### 5.2. LCL161

LCL161 (Novartis) is a structural analogue of the SM compound LBW242 and has also progressed into the clinic [82,83]. It is an orally available monovalent compound that inhibits multiple IAPs including XIAP, cIAP1 and cIAP2 [82,83,84]. *K*_i_ values for individual IAPs do not appear to have been published for this compound. However, like birinapant, it promotes degradation of cIAP1 and appears to have greater activity against cIAP1 and cIAP2 than XIAP [85]. Initial studies with LCL161 showed its efficacy as a single agent towards mutant FLT3- and BCR-ABL-positive leukemia cells [82]. In human HepatoCellular Carcinoma (HCC) cell lines Hep3B and PLC5, LCL161 had single-agent activity (IC_50_ 10.23 and 19.19 μM respectively) and induced cell death [86]. Further studies have also identified LCL161 single-agent activity in Multiple Myeloma (MM) in vitro and in vivo [72,87]. Although there was a mixed anti-tumor response in LCL161 treated MM cell lines, degradation of cIAP1 and inhibition of XIAP was observed in all cells tested [87]. In the study by Chesi et al., MM cells were resistant in vitro to LCL161 except at doses that were not clinically achievable [72]. Other studies have also reported differential responses of tumors to LCL161 treatment. In particular the Pediatric Preclinical Testing Program (PPTP) screened 23 cell lines in vitro and 46 xenograft models in vivo and reported a variable and limited response to LCL161 within these childhood cancer cell lines [88]. The relationship between TNF expression and treatment sensitivity of PPTP cell lines was investigated by the authors [89]. Although two of the most sensitive tumors (anaplastic large cell lymphoma Karpas-299 cell line and medulloblastoma BT-39 xenograft) showed elevated TNF expression, multiple B-precursor Acute Lymphoblastic Leukemia (ALL) xenografts, which also presented with moderate levels of TNF, were resistant to LCL161 treatment [88]. In a later study, Faye and colleagues identified the dependence of RhabdoMyoSarcoma (RMS) tumors on cIAP1 and observed that LCL161 treatment of mice with established Kym-1 RMS xenograft tumors led to tumor cell death and prolonged survival (mean survival days were 46 vs. 77.69 ± SEM) [90]. Strikingly, if LCL161 treatment was initiated prior to tumor growth, mice did not develop tumors by 120 days, effectively preventing the establishment of disease [90]. However, it should be noted that Kym-1 cells are exquisitely sensitive to SM treatment [20], for example compound A (a preclinical precursor of birinapant) has an IC_50_ of ~50 pmol (unpublished).

The first in-human clinical trial of LCL161 tested the safety and effectiveness of the SM in patients with advanced solid tumors (NCT01098838). After treating 53 patients with a dose range of 10 to 3000 mg of LCL161, the MTD was determined as 1800 mg, administered orally once a week. Higher doses led to AEs of cytokine release syndrome, vomiting, nausea, fatigue and anorexia. No patients had an objective response, with 19% having a best response of stable disease [91]. Degradation of cIAP1 and increased circulating cytokine levels, including TNF, were observed in patients dosed with lower concentrations than the MTD, indicating that the dosage of 1800 mg is sufficient to target IAPs irrespective of individual patient pharmacokinetics. As observed in in vitro testing of cancer cell lines, the lack of efficacy of LCL161 in this study correlated with lack of basal production and insensitivity of tumor cells to TNF. Therefore, future studies with LCL161 should focus on the screening of patients that express and are sensitive to TNF [91].

### 5.3. Debio 1143

Debio 1143 (also known as AT-406) is a monovalent SM that was first described in 2011 and developed by Ascenta Therapeutics. This compound has oral bioavailability and targets cIAP1 > cIAP2 > XIAP with *K*_i_ values of 1.9, 5.1 and 66.4 nM respectively [92]. Initial studies evaluated its single-agent capacity to inhibit cancer cell growth in vitro in more than 100 human cancer cell lines and observed that 15% were sensitive. As a single agent, Debio 1143 inhibited tumor growth in MDA-MB-231 breast cancer xenograft models [92]. Further analysis of Debio 1143 showed it also had single-agent activity in 60% of ovarian cancer cell lines tested in vitro. Intriguingly, 3 out of 5 carboplatin resistant ovarian cell lines were sensitive to single-agent treatment. This finding highlights the ability of SMs to drive cell death through molecular mechanisms independent of classical chemotherapy [93].

Analysis of a mouse xenograft model treated with Debio 1143 showed it was quickly absorbed and distributed after oral administration, and in the lung, blood, kidney and liver it reached maximum serum concentration within 15 min [94]. Debio 1143 progressed into first-in-human Phase I clinical trials in patients with advanced metastatic solid cancer (30 patients) and lymphoma (1 patient) (NCT01078649) [95]. Debio 1143 was administered orally at a dose range of 5 to 900 mg, daily from days 1 to 5, then every 14 days and then later every 21 days. A MTD was not confirmed and AEs experienced included fatigue, nausea and vomiting, with 4 patients withdrawing from the trial [95]. As with birinapant and LCL161, on target activity was seen, with rapid degradation of cIAP1 in tumor tissue and no CRs or PRs were observed. Stable disease as best response was seen in 5 patients. The authors of the study highlight the need for combination approaches and screening of sensitive markers for the clinical progression of IAP inhibitors [95].

### 5.4. GDC Smac-Mimetics

Compounds by Genentech, GDC-0152 and CUDC-427 (also known as GDC-0917 and currently being developed by Curis) were reported in 2012 and 2013 respectively. Both are pan-selective IAP antagonists with *K*_i_ values towards cIAP1, cIAP2, XIAP and ML-IAP less than 60 nM [96,97]. The advantage of CUDC-427 over GDC-0152 is its increased oral bioavailability [97].

Initial studies with single treatment of both compounds demonstrated safety towards healthy mammary epithelial tissue and efficacy in inhibiting tumor growth in MDA-MB-231 breast cancer xenograft models [96,97]. Tchoghandjian and colleagues showed that glioblastoma cell lines and primary human samples all expressed IAP proteins, cIAP1/2, XIAP and ML-IAP, albeit at varying levels [98]. Intriguingly, analysis of two cohorts, totaling 101 primary human glioblastoma samples, indicated that high expression levels of ML-IAP were indicative of worse progression-free and overall survival [98]. Therefore, the authors chose to test the ability of GDC-0152, a SM with high affinity towards ML-IAP, to induce glioblastoma cell death in vitro and in vivo. In vitro treatment with GDC-0152 decreased the expression levels of IAP proteins, including ML-IAP in three out of four glioblastoma cell lines, driving apoptotic cell death. In vivo studies using the sensitive U87MG glioblastoma cell line orthotopically xenografted into mice, showed that GDC-0152 postponed tumor development and increased survival [98]. The fact that GDC-0152 and CUDC-427 potently target ML-IAP indicates that they may offer benefit to patients suffering from cancer where ML-IAP is a biomarker of poor prognosis.

The progression of GDC-0152 and CUDC-427 into human clinical trials followed positive preclinical modelling and simulation exercises to predict in-human safety [97]. Although initial data showed GDC-0152 was tolerated, translation of GDC-0152 into additional human clinical trials has been delayed due to withdrawal of a Phase I trial for reasons unrelated to safety or anti-tumor activity (NCT00977067). Similar to GDC-0152, CUDC-427′s progression in human clinical trials has also been arrested. CUDC-427 was tested in a Phase I trial in 42 patients with advanced solid malignancies to determine its safety profile (NCT01226277) [99]. Patients were treated for two out of three weeks with escalating doses of CUDC-427, starting from 5 mg and advancing to 600 mg. AEs included fatigue, nausea, vomiting and rash, with treatment being discontinued in six patients due to dose limiting toxicities. Although out of the 36 patients evaluable for response, 34 had no objective response, two patients (Mucosa-Associated Lymphoid Tissue (MALT) lymphoma of the stomach and the other a BRCA1 (germline) and platinum-refractory ovarian cancer) showed evidence of a durable CR [99]. The profound response experienced by these two patients warrants further investigation into patient indications to select sensitive candidates. Despite the initial promising results, progression of GDC SMs has halted following a second Phase I trial with CUDC-427 that was discontinued (NCT01908413) [99].

### 5.5. Other Smac-Mimetic Drugs

Numerous other SMs have been developed and undergone pre-clinical assessment. These include APG-1387, HGS1029 (also known as AEG40826) and BI 891065 (also known as BI5).

APG-1387 (Ascentage Pharma) has shown anti-cancer effects as a single agent in vitro and in vivo in nasopharyngeal carcinoma cells and in ovarian cancer cells [100,101,102]. APG-1387 is currently in dose-escalation Phase I/II clinical trials to determine safety, tolerability, pharmacokinetics and anti-cancer activity in patients with advanced solid tumors or hematological malignancies (Table 2) (NCT03386526, ACTRN12614000268640 and CTR20150161) [103,104].

HGS1029 (also known as AEG40826) was developed by Aegera Therapeutics Inc. and subsequently licensed to Human Genome Sciences Inc. for commercial development. Preliminary in vitro studies with HGS1029 have shown it has modest activity as a single agent in four out of eight pancreatic cancer cell lines tested [105]. The safety and efficacy of HGS1029 was assessed in a Phase I clinical trial in 44 patients with advanced solid tumors (NCT00708006). The most common AEs were nausea, anorexia, pyrexia, vomiting, diarrhea, fatigue and rash, with dose limiting toxicities being observed in one out of nine patients at 1.4 mg/m^2^ and in two out of six patients at 4.8 mg/m^2^. Best responses were one colon cancer patient presenting with tumor regression and two patients, with Non-Small-Cell Lung Carcinoma (NSCLC) and adrenocortical carcinoma, having stable disease for more than 6 months [106,107]. Despite these promising results, progression of HGS1029 has been attenuated due to termination of a second Phase I clinical trial (NCT01013818).

BI 891065 (also known as BI5) was developed by Boehringer Ingelheim and has higher selectivity towards cIAP1 and cIAP2 compared to XIAP. BI 891065 has shown modest single-agent efficacy in MBT-2 bladder cancer and EMT6 breast cancer cell lines [108]. It is currently in human clinical Phase I trials to determine safety, tolerability and efficacy (Table 2) (NCT03697304, NCT03166631 and NCT04138823).

## 6. Mechanisms of Resistance to Smac-Mimetics and Strategies to Overcome Them

The Phase I/II human clinical trials of SMs indicated they are tolerated as single agents but have low efficacy. Therefore, pre-clinical and clinical research has been conducted to identify biomarkers of response and combination treatments that can increase the effectiveness of SM treatment.

### 6.1. Importance of TNF

The ability of a tumor to produce and respond to TNF (or another TNFSF death ligand) is vital for the anti-tumor effect of SMs, and tumors that lack either of these functions will most likely be resistant to SM treatment [17,20,109,110]. For this reason, an obvious initial combination therapy was the addition of exogenous TNF (or TRAIL) to overcome SM treatment resistance. The efficacy of TNF and/or TRAIL in combination with birinapant is demonstrated by the sensitization of 41 out of 93 birinapant resistant malignancies in vitro [67]. In HNSCC cell lines with differential expression of FADD and cIAP1, addition of TNF or TRAIL dramatically sensitized all tumors to birinapant-mediated killing [76]. Similar results have also been observed in melanoma cell lines where 9 out of 16 birinapant resistant tumors were dramatically sensitized to birinapant-mediated killing with the addition of TNF [68].

Despite these results indicating addition of exogenous TNF in vitro is able to sensitize SM resistant tumors to treatment, administrating TNF systemically to patients is not feasible due to extreme toxicities observed at therapeutically relevant doses [111]. Isolated Limb Perfusion (ILP) is one technique that has been used to administer TNF at therapeutically relevant doses in combination with chemotherapies. However, the technical challenges and innate limitations of ILP means it is only plausible for a minority of localized cancers and therefore alternative methods have been developed to be used in combination with SMs [112]. For example, to overcome the barrier of systemically safe, tumor specific TNF delivery, Yuan and colleagues developed a novel system whereby systemic delivery of Adeno-Associated Virus bacterioPhage-TNF (AAVP-TNF) enables tumor vasculature-targeted gene therapy [113]. This system allows delivery of TNF directly to the tumor tissue, minimizing systemic toxicity [113,114,115]. Co-administration of AAVP-TNF and LCL161 to M21 human xenograft mice led to increased expression of TNF specifically in tumor tissue, and not in healthy organs. Combination therapy was synergistic and significantly prolonged survival of mice [113]. Similarly, cytokine-engineered oncolytic viruses, such as the TNF-armed attenuated oncolytic Vesicular Stomatitis Virus (VSV∆51), combined with the SM LCL161, slowed tumor growth and improved survival rates in mouse models of solid tumors [116]. These findings support the hypothesis that increasing TNF expression in vivo potentiates SM treatment.

Another approach has been to enhance the levels of TNF expressed by the tumor, by targeting parallel signaling pathways. A boutique screen of kinase inhibitors in macrophages showed, surprisingly, that 11 distinct p38 MAPK inhibitors synergized with compound A, the preclinical precursor of birinapant, to increase TNF production and macrophage killing [117]. One of these, LY2228820 (Ralimetinib), was shown to increase induction of TNF by SM treatment leading to synergistic potentiation of birinapant killing of AML cells both in vitro and in vivo [117]. Another approach has been to induce TNF in tumors more conventionally using Toll-Like Receptor (TLR) ligands, such as CpG and poly(I:C). Surprisingly, when combined with LCL161 in an in vivo model, peritoneal injection of poly(I:C) was better at curing the mice than intra-tumoral injection. However, when combined with CpG, the best responses were dual intra-tumoral and peritoneal injections [71]. These results certainly suggest that SMs can combine with circulating TNF and not just TNF produced in the tumor micro-environment.

### 6.2. Combination with Radiation

Having established the neccessicity of TNF for SM-mediated killing, and the potential for increased TNF to overcome treatment resistance, novel combination therapies were explored that combined SMs with TNF enhancing therapy. Hallahan and colleagues reported that treatment of human sarcoma cells with ionizing radiation led to an increase in TNF mRNA and an increased production of TNF protein. The increased production of TNF enhanced radiation-mediated killing through autocrine and paracrine mechanisms [118]. Armed with this knowledge, the combination of SMs with radiation was explored to overcome TNF-mediated SM resistance in cancer. Birinapant or radiation single-agent treatment only modestly extended the survival of mice burdened with FADD overexpressing HNSCC UM-SCC-46 xenograft tumors [74]. Strikingly, however, the combination of SM with radiation cured these mice of HNSCC tumors with no signs of relapse, up to 130 days [74]. A potent increase in endogenous TNF levels in the tumors was found, corroborating the hypothesis that the radiosensitization effect of birinapant is due to an enhancement of TNF in the environment. Similar findings were observed in Esophageal Squamous Carcinoma (ESCC) cells, where the radiosensitizing effect of LCL161 was investigated. ESCC cells were differentially sensitive to SM single-agent treatment. However, addition of radiation increased radiation-induced TNF, DNA fragmentation and apoptosis of these cells. The pan-caspase inhibitor zVAD-FMK attenuated apoptosis, therefore the sensitization mediated by the addition of LCL161 was due to the activation of the TNFR1 extrinsic apoptotic pathway [119]. Further studies showed that Debio 1143 significantly enhanced radiosensitization in NSCLC and HNSCC tumors in vitro and in vivo [120,121]. This sensitization was driven by an increase in autocrine TNF production and cell death was mediated by caspases [120,121]. Due to these promising findings, radiation therapy is being trialed in HNSCC tumors in combination with birinapant (NCT03803774) and Debio 1143 (NCT02022098).

### 6.3. Combination with Chemotherapy

Upon exposure of cells to cytotoxic drugs and DNA-damaging agents, a measurable decrease in endogenous Smac within the mitochondria and an accumulation within the cytosol can be observed [122]. Therefore, SMs provide a means to augment the natural response to cytotoxic compounds. Chemotherapy remains the front-line treatment for a range of cancers, rationalizing the exploration of pairing SMs with chemotherapies for combination treatment. Paclitaxel is one of the first-line chemotherapy treatments for NSCLC. However, due to its limited efficacy in some patients, new combination treatments are being investigated [123]. An increase in expression of cIAP levels has been shown to correlate with poor prognosis and lower overall survival in various types of cancer, including NSCLC [124,125,126,127]. Therefore, combining the SM LCL161 with paclitaxel in NSCLC tumors was investigated. Addition of LCL161 to paclitaxel therapy increased TNF expression, degradation of cIAP1/2 and activation of caspase-8 dependent apoptotic signaling, sensitizing NSCLC cancer cells to treatment in vitro [124]. Similar findings were observed in mice xenografted with NSCLC tumors where LCL161 plus paclitaxel treatment had better anti-tumor activity than either treatment alone [124].

Treatment of HNSCC cell lines with birinapant plus docetaxel was more effective than either treatment alone in vitro [76]. However, while birinapant plus docetaxel treatment of mice burdened with HNSCC xenografts significantly reduced tumor volume, there was no extension in survival compared to control treated mice [76]. Surprisingly, however, there was a significant extension in survival with birinapant single-agent treatment, although it was less effective at reducing tumor volume [76]. The authors suggested that a different dosing schedule might have increased survival in the combination treated animals, but regardless, the results yet again emphasize that in vivo response to birinapant can be better than predicted from in vitro studies. In HL-60, OVCAR-3 and HT-1376 cancer cell lines, the effects of birinapant treatment could be enhanced by the addition of chemotherapy agents SN-38 (active metabolite of irinotecan), gemcitabine or 5-azacytidine, but not pemetrexed, vemurafenib, bendamustine or sorafenib [67]. Interestingly, in the HT-1376 bladder cancer cell line, the potentiation of birinapant and gemcitabine treatment was not attenuated by co-treatment with an anti-TNF antibody, thus indicating that the increase in cell death was via a TNF-independent-mechanism in this tumor [67].

Platinum-based chemotherapy, commonly carboplatin, is the front-line therapy for ovarian cancer patients. However, patients can develop resistance to treatment [128,129]. In High-Grade Serous ovarian Cancer (HGSC) primary samples, a small proportion of cells were platinum resistant and possessed stem cell characteristics of tumor initiation, multi lineage differentiation, self-renewal and had high expression of IAP proteins. Co-treatment of birinapant with carboplatin led to sensitization of these cells and increased killing in a caspase-8 dependent mechanism in vitro and in xenograft HGSC models [130]. As expected, Human Ovarian AdenoCarcinoma (HOAC) cells had a variable response to carboplatin single-agent treatment, with three out of five being resistant. However, despite resistance to chemotherapy, co-treatment with Debio 1143 with carboplatin sensitized these cells to cell death in vitro [131]. Treatment of carboplatin and Debio 1143 in vitro resistant SKOV-3 HOAC xenograft burdened mice with carboplatin had no effect, whilst treatment with Debio 1143 single agent induced a slow-down in tumor growth and complete regression in one out of seven mice. This effect was potentiated with the addition of carboplatin leading to slow-down of tumor growth in two mice and complete regression in five mice (out of seven) [131]. Furthermore, in vivo treatment of OVCAR3ip (cells selected in vivo from OVCAR3 parental cells to form ascites) carboplatin-resistant ovarian xenograft models with Debio 1143 in combination with carboplatin was able to prolong survival of mice better than single treatments [93]. The capacity of SMs to act as single agents or in combination with carboplatin to kill carboplatin resistant ovarian cancer cell lines validates them as a combination or alternative therapy to overcome resistance [93].

The preclinical data discussed above indicated SMs are more efficacious when combined with TNF inducing chemotherapies than alone. For this reason, birinapant was combined with several chemotherapies including, carboplatin/paclitaxel, irinotecan, docetaxel, gemcitabine or liposomal doxorubicin for the treatment of patients with solid tumors. Co-treatment of birinapant with these diverse chemotherapies in 124 patients with refractory/relapsed solid tumors did not limit the dose of chemotherapy administered. Despite seven patients experiencing reversible Bell’s palsy symptoms, overall birinapant was well tolerated in combination with chemotherapy as a treatment. Clinical benefit was observed in numerous patients, with 11 patients having a PR and 61 having stable disease. Of the chemotherapies tested, irinotecan enhanced birinapant’s activity the most, even in patients that had previously failed irinotecan therapy (NCT01188499) [132]. Therefore, a Phase II extension study was conducted combining birinapant with irinotecan in irinotecan-relapsed or refractory metastatic ColoRectal Cancer (CRC) patients [133]. Birinapant was administered at a fixed dose or in an Ascending Dose Schedule (ADS) in combination with irinotecan at a fixed dose. The combination was well tolerated, and the ADS appeared to prevent symptoms of Bell’s palsy. Two patients achieved a PR, while 27 had stable disease. Together, this study supports the idea that combining birinapant with the TNF inducing chemotherapy irinotecan may be a feasible therapeutic strategy for irinotecan resistant tumors (NCT01188499) [133].

As discussed above, the chemotherapy paclitaxel has been shown to potentiate LCL161 mediated killing in solid tumors, including Triple Negative Breast Cancer (TNBC) [124,134,135,136]. Phase II clinical trials were initiated [137], following Phase I trials that indicated that LCL161 plus paclitaxel therapy is well tolerated [134]. Interestingly, for this study the TNF-based Gene expression Signature (GS) was determined for each patient and used as a predictor of sensitivity to SM-mediated cell death [137]. Bardia and colleagues conducted a global trial incorporating molecular pre-screening to investigate the neoadjuvant treatment of LCL161 and paclitaxel in TNBC patients assigned as GS-positive (more likely to respond to SM treatment) vs. GS-negative (less likely to respond) [137]. Of 207 patients, 30.4% had a GS-positive score and combination treatment was more effective than paclitaxel alone treatment. However, in the GS-negative group comprising 69.6% of the patient population, there was an antagonistic effect in combination treatment compared to control arms. This study highlights the importance of molecular screening to determine eligibility of patients and the analysis of possible increased toxicities (NCT01617668) [137].

### 6.4. Combination with Bcl-2 Inhibitors

B-cell lymphoma 2 (Bcl-2) prevents Bax/Bak mediated disruption of the mitochondrial outer-membrane, preventing cell death and efflux of cytochrome *c* from the mitochondrial inter-membrane space [138,139,140]. Efflux of endogenous Smac from within the mitochondria is also regulated by Bcl-2 and cells overexpressing Bcl-2 inhibit the release of Smac from the mitochondria following apoptotic stimulus [37,122]. Combining SMs with other specific inducers of cell death, such as Bcl-2 inhibitors, might increase efficacy and reduce toxicity. Preliminary studies where the authors knocked down Bcl-2 which led to resistant Huh7 cells becoming sensitized to LCL161 treatment in vitro, were nevertheless discouraging because the level of cell death achieved was minimal, less than 20% [86]. More impressive results were obtained combining the putative Bcl-2 inhibitor SC-2001 (a derivative of obatoclax) with LCL161 to treat Huh-7 xenograft tumors in vivo [86]. MM cells have been shown to have high expression of anti-apoptotic Bcl-2 family members [141,142] and IAP family members [143,144], suggesting that the co-inhibition of these two families of proteins may be beneficial for the treatment of MM. Co-treatment with obatoclax and LCL161 led to a synergistic killing of MM cell lines [145]. However, this synergistic killing may not be due specifically to obatoclax inhibiting Bcl-2 because a number of well controlled studies have shown that obatoclax kills cells in a Bax-Bak independent manner and does not act as a BH3 mimetic [146,147]. A more recent study combining the specific Bcl-2 inhibitor ABT-199 with SMs birinapant or Debio 1143 showed an increase in human colon adenocarcinoma cell death compared to single-agent treatments [148]. Together, these preclinical studies indicate the potential for targeting the intrinsic and extrinsic apoptosis pathways in SM combination therapy.

### 6.5. Combination with Immunotherapy

Immunotherapy harnesses the immune system to kill tumors. Kearney et al. 2017 showed that the SM birinapant sensitized tumor cells to TNF dependent killing by Cytotoxic Lymphocytes (CLs), both CD8+ T cells and Natural Killer (NK) cells. Upon antigen recognition or NK-activating receptor activation, CLs naturally respond by inducing TNF. Surprisingly, given the data showing the ability of SMs to increase TNF levels, birinapant did not increase T-cell production of TNF [149]. On the other hand, tumor-derived Programmed Death-Ligand 1 (PD-L1) engagement of its receptor, Programmed cell Death protein 1 (PD-1), expressed on CLs, decreased CL production of TNF. Furthermore, while birinapant did not increase TNF secretion by CLs, it did sensitize the tumor cells to TNF induced death. Together, these results suggested that the combination of the Immune Checkpoint Inhibitor (ICI), anti-PD1, and birinapant would be a very effective way to increase CL killing. And indeed, this is what the authors observed [149]. Similarly, Beug and colleagues in an extensive and very detailed study, showed that combining the ICIs, anti-PD1 or anti-Cytotoxic T-Lymphocyte-Associated protein 4 (anti-CTLA-4), with the SM LCL161 greatly increased survival in intra-cranial mouse glioblastoma models and produced durable cures [150]. These results are particularly significant on several levels. Firstly, they show that the combination therapy works well in vivo without any reported toxicity. Secondly, the SM was delivered orally, yet the blood brain barrier, a significant barrier for many drugs, was not an impediment, and thus the combination works in one of the most challenging in vivo environments. Thirdly, the authors showed that more than one SM and ICI cocktail was effective, boosting confidence in the general utility of the approach. Lastly, the durable response was associated with immunological memory suggesting the potential of the therapy to deliver long-term cures. As in single-agent studies, TNF was an important part of the cytotoxic response and also required CD8+ T-cells [150]. Encouragingly, an independent study with the SM BI 891065 in combination with an anti-PD1 antibody also eradicated breast cancer tumors in immunocompetent mice [108].

Clinical trials in solid tumors with diverse SMs and immunotherapy are currently in progress; birinapant and Pembrolizumab (NCT02587962), LCL161 and PDR001 (NCT03111992 and NCT02890069), Debio 1143 and Nivolumab, Pembrolizumab or Avelumab (NCT04122625, NCT03871959 and NCT03270176), and BI 891065 and BI 754091 (NCT03697304, NCT03166631 and NCT04138823) (Table 2).

An alternative option to amplifying the immune response, is to reduce the threshold of a tumor cell to respond to immunotherapy. Using a CRISPR/Cas9 screen, Kearney et al. 2018, showed that the suppression of TNF, Interferon (IFNγ) and antigen presentation were key mechanisms by which tumors can evade the attack of CLs [151]. This matched well with previous clinical data showing that IFNγ plays an important role in the efficacy of ICIs [152], but for the first time suggested TNF signaling was also an important component. They found that TNF was a potent NK cell effector molecule and that TNF-mediated apoptosis is important in a NK cell attack [151]. Subsequently, Vredevoogd and colleagues combined these earlier observations to show that increasing sensitivity of tumor cells to TNF killing by removal of TRAF2 enhanced the therapeutic effect of ICI drugs. And, as expected, given the important role TRAF2 plays in recruiting cIAPs and the synergistic effects of combining birinapant with ICIs in vitro shown by Kearney and colleagues, inhibition of cIAP1/2 by birinapant led to an even stronger response when combined with ICI drugs (anti-PD-1 therapy) [153]. Work showing that SMs can also synergize with Chimeric Antigen Receptor (CAR) T-cell therapy is discussed in an accompanying review in this series [73,154].

### 6.6. Inducing Necroptosis

SMs can sensitize cells to both apoptotic and necroptotic cell death pathways mediated by TNFR1. As previously discussed, the majority of current cancer chemotherapies utilize the intrinisic apoptotic pathway to induce cell death. As many cancers have evolved resistance to cell death via apoptosis, the ability of SMs to regulate the TNF cell death pathways through promoting both apoptosis and necroptosis provides a promosing novel therapeutic avenue towards resistant malignancies [155,156]. Proof of principle for this concept has been shown as an effective way to kill AML cells both in vitro [157] and also safely in vivo [7]. Specifically Brumatti and colleagues induced necroptosis by combining birinapant with the U.S Food and Drug Administration (FDA) approved caspase inhibitor IDN-6556 (Emricasan) [7]. However, SM-mediated necroptotic cell death does not always require pharmaceutical inhibition of caspases. Birinapant has been observed to mediate cell death through dual action of apoptotic and necroptotic mechanisms in ALL [158]. Similarily, addition of Debio 1143 with the chemotherapy carboplatin led to ovarian cancer cell apoptotic or necroptotic cell death, depending on the cell line [131]. Reactive Oxygen Species (ROS) are volatile molecules and high levels can induce programmed cell death in their own right [1,159]. They have also been shown to influence cellular responses to TNF by regulating the NF-κB and apoptotic pathways [160]. In some cases, the presence of ROS has been shown to enhance both SM-mediated apoptotic and necroptotic cell death [8,77,161]. Taken together, these studies suggest that birinapant may possess a dual activity (apoptotic and non-apoptotic) in cancer cells, and that the ability to promote non-apoptotic cell death makes it an attractive drug for the treatment of apoptotic resistant cancers.

### 6.7. Mechanical Resistance

Recent studies have shown that SMs, including the clinically relevant birinapant, are substrates for the MultiDrug Resistance 1 (MDR1; gene name ABCB1) pump, also known as P-glycoprotein (P-gp) [162,163] (Morrish et al., unpublished). These transporters have been well characterized and their substrates are many. However, these recent studies identifying SMs as substrates have the potential to further inform future clinical trials in using patient MDR1 profiles for targeted therapy.

## 7. Future Directions and Conclusions

In this review, we have highlighted the progression of SMs from bench to bed-side. Eight compounds have been tested in humans, and all were well tolerated, had a reasonable safety profile, were shown to hit their target and displayed some anti-tumor activity. SMs are inherently non-toxic towards healthy tissue and the reason for this is not entirely understood. It has been proposed that because SMs are targeted drugs and specifically affect IAP signaling pathways, they have less toxicity than chemotherapies which alter many pathways [164,165,166,167]. Another explanation might be that abnormal expression of IAPs and/or TNF by the tumor or stromal cells in its environment leads to addiction to NF-κB and/or TNFR1 signaling pathways. Following the preliminary success of the SM compounds in vitro and in vivo, and their apparent low toxicity towards healthy tissue, they progressed into in-human clinical trials as single agents. However, a common draw back for all compounds is their limited clinical activity as single agents. Extensive investigation has been conducted over the last decade to enhance the efficacy of SMs as anti-cancer therapy. The strengths and weaknesses of SM therapy are complex and intertwined. TNF is an essential component for SM-mediated cell death. Tumors that do not produce and respond to TNF are inherently resistant to SM treatment, limiting therapy, but unfortunately it is not feasible to directly co-administer TNF to patients. Therefore, various combinations have been investigated to safely enhance the levels of TNF experienced by the tumor by targeting other signaling pathways, or using chemo-, radiation or immunotherapy. Alternative strategies to increase SM potential are through additional targeting of the intrinsic apoptosis pathway, activating the alternative cell death pathways such as necroptosis or preventing mechanical efflux of SM drugs. A common thread running through all these treatment options is that they will not work effectively in all patients, and thus the identification and validation of biomarkers of response are required to ensure that patients receive the most effective SM combination therapy. While there remains much work to be done, we believe the very specific action of SM drugs, with little evidence of off-target activity, the good safety profile (probably linked to specificity) and the potential to synergize with other equally exciting up-coming treatments, such as immunotherapy, augurs well for the clinical future of SMs.

## Figures and Tables

**Table 1:** *K*_i_ (nM) values for clinically relevant Smac-Mimetic compounds.

Smac-Mimetic	cIAP1	cIAP2	XIAP	ML-IAP	Reference
Birinapant	~1	36	50 ± 23	~1	Condon et al., 2014
LCL161	−	−	−	−	Derakhshan et al., 2016
Debio 1143	1.9	5.1	66.4	−	Cai et al., 2011
GDC-0152	17	43	28	14	Flygare et al., 2013
CUDC-427	<60	<60	<60	<60	Wong et al., 2013

**Table 2:** In progress clinical Smac-Mimetic trials.

Smac-Mimetic	Adjuvant Therapy	Cancer	Phase	Clinical Trial	Date
Birinapant	Pembrolizumab	Solid cancer	I/II	NCT02587962	Aug-17
Birinapant	Radiation	HNSCC	I	NCT03803774	Jan-19
LCL161	None	Myelofibrosis	II	NCT02098161	Dec-14
LCL161	Immunotherapy ^a^	Solid tumors ^b^	Ib	NCT02890069	Oct-16
LCL161	Immunotherapy ^c^	Multiple myeloma	I	NCT03111992	Dec-17
LCL161	Topotecan	Solid tumors ^d^	I/II	NCT02649673	Mar-16
Debio 1143	Nivolumab	Solid cancer	I/II	NCT04122625	Apr-19
Debio 1143	Pembrolizumab	Solid tumors ^e^	I	NCT03871959	Sep-19
Debio 1143	Avelumab	NSCLC	Ib	NCT03270176	Oct-17
Debio 1143	Cisplatin/radiotherapy	HNSCC	I/II	NCT02022098	Oct-13
APG-1387	None	Solid cancer/Hema	I/II	NCT03386526	Nov-17
BI 891065	Immunotherapy ^f^	Solid tumors ^g^	I	NCT03166631	Sep-17
BI 891065	Immunotherapy ^f^	Neoplasm metastasis	II	NCT03697304	Mar-19
BI 891065	Immunotherapy ^f^	Neoplasm	I	NCT04138823	Nov-19

^a^ PDR001 checkpoint inhibitor. ^b^ Colorectal cancer, non-small cell lung carcinoma (NSCLC), triple negative breast cancer, renal cell carcinoma. ^c^ PDR001, anti-IL-17 monoclonal antibody CJM112. ^d^ Small cell lung cancer, ovarian cancer. ^e^ Adenocarcinoma of the pancreas, colon and rectum. ^f^ anti-PD-1 monoclonal antibody BI 754091. ^g^ Neoplasms, neoplasm metastasis, NSCLC.
